# Free Non-Vascularized Fibular Graft for Treatment of Pediatric Traumatic Radial Bone Loss: A Case Report

**DOI:** 10.5704/MOJ.1307.003

**Published:** 2013-07

**Authors:** K Thevarajan, PC Teo

**Affiliations:** Department of Orthopaedics, Sultanah Aminah Hospital, Johor Bahru, Malaysia; Department of Orthopaedics and Traumatology, Universiti Kebangsaan Malaysia, Kuala Lumpur, Malaysia

## Abstract

Various methods, such as vascularized bone transfers, Illizarov bone transport, allogenic bone grafts, bone graft substitutes, are available in treating traumatic bone loss. Free non-vascularised fibular graft is an autografting method that only requires minimal facilities or expertise. However, this method is not popularized due to its avascular property and there is not many reports regarding its use in treating a large traumatic bone loss. We reported a case in our center to demonstrate its possibility of successfully treating the traumatic radial bone loss in pediatric patient. Patient had good recovery with the regain of good range of movement of forearm and there is no harvest site morbidity after two years of follow up.

**Key Words:**

Free non-vascularized fibular graft, pediatric radial bone loss

## Introduction

Traumatic bone loss poses a challenging problem for orthopaedic surgeon. Many surgical interventions are available for bridging bone defects: bone grafting, free vascularised fibular grafts, or bone transport with an Illizarov fixator. Above all, free microvascular bone grafts have been popularised due to its advantages in bone healing and fighting infection. However difficult technique and infrequently available microsurgical facilities in Malaysia makes the non-vascularized cortical autograft to be the reasonable mode of treatment. This case report is studying a pediatric patient who was treated successfully with nonvascularized fibular autograft for radial bone defect.

## Case Report

An 11-year-old girl, no known medical illness, sustained open fracture of the right radius and ulna. She was treated with wound debridement and plating of the right radius. Unfortunately, it was complicated with non-union of the radius secondary to infection after 2 months. Infected implant and 5 cm sequestrum was removed from radius bone.

Infection was successfully controlled. Patient was electively admitted after 3 months for corrective surgery. Intraoperatively, the radial ulnar area was clean and there was no evidence of infection. Tissue was taken from the same area for culture and sensitivity. The result showed no growth. The radial graft area was prepared by excising both the proximal and distal end of radius by using an oscillating saw. The proximal end was a clean cut and the medullary canal was clearly identified, the distal end was cancellous bone and there was no medullary canal hence the graft was then placed in acceptable anatomical position with good contact of both ends. Not much soft tissue dissection was done in that area, only around the bone end for adequate exposure, so as to preserve viable tissue in that area.

Seven cm right fibula graft was harvested meticulously with preservation of the periosteum. Non-vascularized fibular was then grafted at the radius. Osteotomy of right ulna was done to shorten the ulna bone for reduction of the distal radialulnar joint. Keeping in mind with the history of infection, the ulna bone was shortened with minimal approach and left undisturbed without internal fixation. By understanding the potential of remodelling as well, the ulna was left with bayonet apposition hence the ulnar was still being in slight angulation. The stable fixation was achieved with intramedullary k wires of the radius and fibular graft plus kwire of the distal radial-ulnar joint. The fixation was then protected with forearm full length cast.

Recovery was uneventful after two years of follow up. There is no gross angular deformity of the right forearm at the final follow up. Her fibular graft was incorporated into radius bone. Ulna bone was united with good remodelling. Regeneration of fibula was even noted at the donor site.

Range of movement of right forearm was almost full with no difficulty in daily activities. Her right wrist’s flexion is 0-90 degrees, extension is 0-75 degrees, ulna and radial deviation is normal, supination is full and pronation is 0-45 degrees. Her right elbow has full range of motion.

Her gait is normal and she is able to squat with ease. There is no pain at the donor area. Range of motion of the right knee and ankle are full. Both the lower limbs are equal in length. There is no limb length discrepancy noted.

Currently, she plays hockey for the school. Her physical appearance of the right forearm and leg appears normal. Patient is satisfied with the outcome.

## Discussion

Free non-vascularized fibular graft for treatment of posttraumatic bone defects had been well studied. Study^1^ suggested to harvest fibula subperiosteally and held by some kind of fixation of the fibular strut ends to the ends of the bone defect. In order to maintain donor site ankle instability, fibula graft should be harvested by preserving at least 6-8 cm of the residual length distally.

However, patient selection is the key to success in nonvascularized fibula grafting. The rate of healing declines with increasing age up to skeletal maturity, but after completion of skeletal growth, the rate of fracture healing does not appear to decline significantly with increasing age, nor does the risk of non-unions significantly increase. It should be avoided when the recipient bed is not ideal like atrophic fracture non-union and post-traumatic infective non-unions. It yield higher union percentage if used in upper limb as compared to use in lower limb^2^.

The reported union rate for traumatic large bone defect in nonvascularised fibula grafting is almost 90%^1^,^2^. One of the report showed their graft incorporation was 80% in all treated patients with average defect length of 6.5cm^3^. Beside traumatic cases, the use of non-vascularized fibula grafting is applied in giant cell tumour reconstruction surgery. The result is quite promising too^4^.

The disadvantage of fibula grafting is the donar site complications such as donar site infection, ankle instability, ankle valgus and rare tibia stress fracture.

In our case report, the good result happened in the 11 year-old child with complete union of the non-vascularised fibula graft and almost complete range of motion of the forearm. Surprisingly, the regeneration of fibula in donar site was observed as well. One study actually showed the possibility of this regeneration in pediatric cases^5^.

As a conclusion, the non-vascularised fibula grafting is an acceptable method of treating the long bone defect or limb salvage procedure of paediatric cases in the center that lack of advanced facility.

**Fig. 1:**
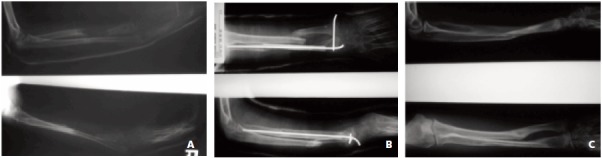
: The progression of an 11 year-old child who was treated with non-vascularized fibula graft at her radial bone defect. Picture A shows the large bone loss at radius after debridement for infection. Picture B shows the non-vascularized fibula grafting after infection controlled. The fibula was held with intramedullary k-wire. Picture C shows union of the fibula graft with remodelling of the radius and ulna after two years.

**Fig. 2:**
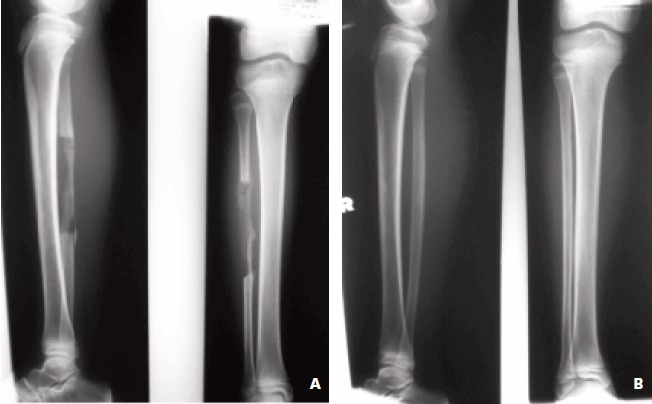
: X-ray of the donar fibula site. Picture A shows sign regeneration of fibula at the donar site after few months. Picture B shows full regeneration of fibula after two years.

**Fig. 3:**
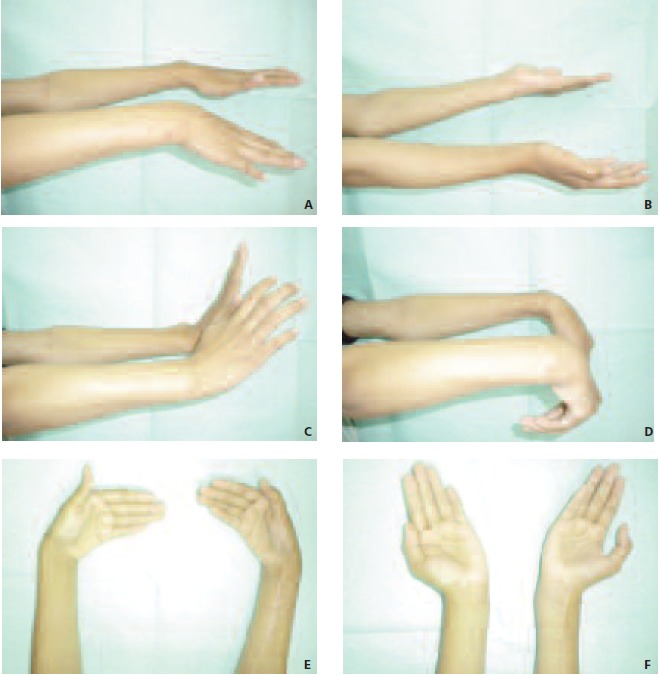
: Range of movement of forearm and wrist is almost full after two years of fibula grafting. Patient has no difficulty in performing supination, pronation, flexion, extension, abduction and adduction.
